# Clinical application of 3D-printed fusion cage implantation in treating cervical spondylotic myelopathy

**DOI:** 10.12669/pjms.40.9.9125

**Published:** 2024-10

**Authors:** Yue Ma, De-bao Zhang, Fan Li, Yang Song

**Affiliations:** 1 Yue Ma, Department of Orthopaedics, Affiliated Hospital of Beihua University, 132011 Jilin, P. R. China; 2 De-bao Zhang, Department of Orthopaedics, Affiliated Hospital of Beihua University, 132011 Jilin, P. R. China; 3 Fan Li, Department of Orthopaedics, Affiliated Hospital of Beihua University, 132011 Jilin, P. R. China; 4 Yang Song, Department of Orthopaedics, Affiliated Hospital of Beihua University, 132011 Jilin, P. R. China

**Keywords:** 3D-Printed, Fusion Cage, Cervical Spondylotic Myelopathy, Intervertebral Space Height, Cervical Cobb Angle

## Abstract

**Objective:**

To investigate the clinical efficacy of 3D-printed fusion cage implantation in treating cervical spondylotic myelopathy.

**Methods:**

A retrospective analysis was performed on 52 patients with single-segment cervical spondylotic myelopathy who received anterior cervical discectomy and fusion in Affiliated Hospital of Beihua University from July 2019 to July 2022. They were divided into the 3D group and the control group. Afterward, the perioperative indicators were compared between the two groups. Meanwhile, the Japanese Orthopaedic Association (JOA) scores, Visual analogue scale (VAS) and neck disability index (NDI) were recorded pre-operation and one-year post-operation. Evaluate the fusion rate of two group of intervertebral fusion cages.

**Results:**

The JOA score and NDI of the two groups of patients were significantly improved compared to pre-operation(P<0.05), and the JOA score, JOA score improvement rate, and NDI of the 3D group were better than the control group (P<0.05), the VAS scores were significantly improved compared to pre-operation(P<0.05), but there was no statistically significant difference between the groups (p >0.05). The intervertebral height, angle, and cervical Cobb angle of the two groups of patients were significantly improved compared to pre-operation (P<0.05), and the improvement of intervertebral height, angle, and cervical Cobb angle in the 3D group was better than the control group(P<0.05).

**Conclusion:**

The clinical efficacy of 3D printed intervertebral fusion cage placement in the treatment of cervical spondylotic myelopathy may be reliable, and it would be better than traditional intervertebral fusion cages in maintaining the height, angle, cervical Cobb angle, and fusion rate of the intervertebral space.

## INTRODUCTION

Cervical spondylosis has seen a significantly increasing incidence in recent years.^1^,^2^ As a common type of cervical spondylosis in clinical practice, cervical spondylotic myelopathy (CSM) is difficult to treat, and patients are often subject to poor prognosis.^3^,^4^ Therefore, once clinically diagnosed with CSM, if conservative treatment is ineffective in the short term, surgical treatment should be performed as soon as possible. Which anterior cervical discectomy and fusion (ACDF) is a clinically validated classic surgical method and has been considered the “gold standard” for treating CSM.^5^

The principle of ACDF is to directly decompress the compressed nerves and reconstruct the stability of the cervical spine through intervertebral fusion, with its surgical efficacy closely related to the fusion cage. Currently, the most widely used fusion cage in clinical practice is the polyetheretherketone cage (PEEK Cage), which, despite its wide application, also comes with disadvantages, such as poor fusion, and stress shielding.^6^ 3D printing, as an emerging technology widely used in various industries worldwide has been increasingly applied in clinical practice over the past few years. The 3D-printed fusion cage (3D Cage) has a porous structure and can provide a large rough contact surface, thus facilitating the ingrowth of bone. In this paper, a retrospective analysis is performed on the case data of CSM patients treated with ACDF using 3D Cage in Affiliated Hospital of Beihua University while also exploring the clinical application effect of 3D Cage in the surgical treatment of CSM.

## METHODS

This was a retrospective study. A total of 52 cervical spondylotic myelopathy (CSM) patients treated with ACDF in Affiliated Hospital of Beihua University from July 2019 to July 2022 were selected and divided into the 3D group (n=25; treated with 3D Cage) and the control group (n=27; treated with PEEK Cage) following their preference.

### Ethical Approval:

The study was approved by the Institutional Ethics Committee of Affiliated Hospital of Beihua University (No.:2023025; date: April 18,2023), and written informed consent was obtained from all participants.

### Inclusion Criteria:

- Patients who met the diagnostic of CSM.^3^
- Patients with single-segment lesions and symptoms of cervical spinal cord injury.
- Patients whose clinical symptoms didn’t improve, or even worsened after receiving conservative treatment for over three months.
- Patients with complete medical records and imaging data.
- Patients who received ACDF treatment.

### Exclusion Criteria:

- Patients with cervical spinal cord injury due to other reasons.
- Patients who met the diagnostic criteria of CSM but suffered multi-segment lesions.
- Patients with spinal cord occupying lesions, syringomyelia, and other central nervous system lesions.
- Patients with other serious diseases.

### Surgical Methods and Perioperative Treatment:

The 3D group used the titanium alloy cage produced by Beijing AK Medical Co., Ltd., while the PEEK cage produced by Shandong WEGO ORTHP Co., Ltd. Patients in both groups received ACDF treatment, in which the intervertebral disc tissue of diseased segment was completely removed and the cartilage endplate and hyperplastic osteophyte were scraped off before placing the cage, followed by vertebral fixation with the anterior cervical titanium plate in an appropriate size. Patients were instructed to engage in early activities under the protection of their necks. All the patients were follow-up for one year.

### Observation Indicators:

The length of hospital stays, operation time, intraoperative blood loss, postoperative complications, and fusion were recorded in the two groups. Meanwhile, the JOA, VAS and NDI scores were performed pre-operation and postoperative. The JOA score improvement rate (%) at postoperative was calculated for both groups, i.e., (postoperative JOA score - preoperative JOA score)/(17 - preoperative JOA score)×100%. Moreover, the cervical Cobb angle and intervertebral space height and angle were observed one year post-operation. According to Kandziora’s standards, evaluate the fusion status of the intervertebral fusion cage one year post-operation. Fusion standard: The fusion device has no offset and no transparent band around it;Change in intervertebral space angle of fusion segment on cervical dynamic radiograph<5°;No looseness of the internal fixture. All the surgeries and follow-up work of all patients was completed by the same group of surgeons.

### Statistical Analysis:

Data processing and statistical analysis were performed using SPSS 22.0 software. An independent sample t test was used, enumeration data were expressed as percentages (%), with the χ² test used for comparison. Measurement data were expressed as *χ̅*±*S*, with comparison by t-test. P<0.05 was considered statistically significant.

## RESULTS

No significant differences in general information were found between the two groups (P>0.05) (Table-I). There was no significant difference in hospital stay, operation time, intraoperative blood loss, and postoperative complications between the two groups(P>0.05), (Table-II).

**Table-I:** Comparison of General Data between two Groups.

Item	3D Group (n=25)	Control Group (n=27)	t/²value	P value
Age(years-old)	31-72(59.32±9.43)	30-78(62.11±10.65)	-0.998	0.323
Sex (M/F, n)	16/9	18/9	0.041	0.840
Diseased Segment(n)			0.293	0.961
C_3/4_	8	9		
C_4/5_	9	11		
C_5/6_	5	4		
C_6/7_	3	3		

**Table-II:** Comparison of Perioperative Conditions between two Groups.

Item	3D Group (n=25)	Control Group (n=27)	t/c^2^ value	P value
Hospital Stay(d)	8.76±1.36	8.74±1.26	0.053	0.958
Operation Time(min)	93.20±6.27	96.11±8.70	-1.375	0.175
Intraoperative Blood Loss(ml)	83.2±7.62	88.67±6.50	-1.768	0.083
Complications(n)			2.311	0.510
Dysphagia	1	2		
Wound Infection	0	0		
Screw Loosening	1	2		
Implant Collapse	0	1		
Implant Migration	1	0		

Compared with preoperative values, JOA scores and NDI were significantly improved in both groups at postoperative (P<0.05), the 3D group saw better JOA scores and NDI at postoperative than control group (P<0.05). JOA improvement rate improved by 74.49%±10.22% and 62.10%±10.51% in the 3D group and the control group (t=4.306, P=0.000). At postoperative, the VAS scores of both groups of patients were significantly improved compared to pre-operation(P<0.05), but there was no statistically significant difference between the groups (P>0.05), Table-III.

**Table-III:** Comparison of JOA Scores and NDI Pre-Operation and at the Final Follow-up between two groups.

Group	JOA Scores			NDI (%)		VAS Scores	

	Preoperative (points)	Last Follow-up (points)	Improvement Rate (%)	Preoperative	Last Follow-up	Preoperative (points)	Last Follow-up (points)
3D (n=25)	4.12±0.78	13.72±1.31^a^	74.49±10.22	39.04±2.61	18.64±1.50^a^	7.92±0.81	0.60±0.50
Control (n=27)	4.19±0.79	12.15±1.32^a^	62.10±10.51	38.89±3.99	21.11±2.91^a^	8.11±0.70	0.81±0.62
*t* value	-0.300	4.307	4.306	0.160	-3.800	0.912	1.365
*P* value	0.766	0.000	0.000	0.873	0.000	0.366	0.178

***Note:*** Compared with preoperative values in the same group,

a P < 0.05.

During the postoperative final follow-up, the intervertebral space height, angle and cervical Cobb angle in both groups were significantly increased (P<0.05); the 3D group saw a higher intervertebral space height, angle and a greater cervical Cobb angle than control group(P<0.05). Table-IV. All patients in 3D group had bone fusion at postoperative, with a fusion rate of 100%. However, in control group, four patients did not achieve bone fusion, with a fusion rate of 85.19% (c^2^= 4.012, P=0.045).

**Table-IV:** Comparison of Intervertebral Space Height and Cervical Cobb Angle Pre-operation and at the Final Follow-up between two Groups.

Group	Intervertebral Height(mm)		Intervertebral Angle(mm)		Cervical Cobb Angle (°)	

	Preoperative	Last Follow-up	Preoperative	Last Follow-up	Preoperative	Last Follow-up
3D (n=25)	6.04±0.84	8.44±0.71^a^	7.56±0.88	12.75±0.78^a^	11.24±1.13	17.52±1.23^a^
Control (n=27)	6.04±0.90	7.67±0.83^a^	7.53±1.06	11.31±1.29^a^	11.19±1.04	14.30±1.23^a^
*t* value	0.012	3.587	0.113	4.850	0.182	9.429
*P* value	0.990	0.001	0.911	0.000	0.856	0.000

***Note:*** Compared with preoperative values in the same group,

a P < 0.05.

## DISCUSSION

In this study, the clinical efficacy of 3D Cage and traditional PEEK Cage implantation in CSM patients was compared and analyzed. The JOA scores, JOA improvement rate, NDI and VAS scores were significantly improved in the two groups; the 3D group saw significantly better JOA scores, JOA improvement rate, and NDI than the control group. However, there was no difference in VAS scores between the two groups of patients, indicating that the application of 3D-printed Cage could significantly improve the clinical symptoms of patients and improve the surgical efficacy without increasing patient pain. The possible reason is that 3D Cage can be pre-customized with a well-matched implant model based on the patient’s anatomy data, providing a large contact area and satisfactory matching between the implant and tissue. Meanwhile, a 3D Cage has a porous structure inside, which is beneficial to the ingrowth of new bone and bone fusion, along with a high degree of long-term cervical fusion and better postoperative cervical stability and mobility for patients.

CSM with its pathological basis manifested as neck pain, upper limb numbness, increased lower limb muscle tension, and other symptoms due to anterior compression of the cervical spinal cord.^7^,^8^ If the conservative treatment effect is not satisfactory in clinical practice, timely surgical treatment is necessary.^9^,^10^ Otherwise, it will seriously affect the work and life of patients and may even lead to death in server cases.^11^,^12^ Because ACDF is a direct anterior cervical approach to remove the pressure source, achieving direct decompression,^13^ reconstructing cervical stability, and restoring cervical physiological lordosis^14^, it has the advantages of convenient access, simple operation, and low incidence of surgical complications.^15^

ACDF is a surgical method currently recognized as the “gold standard” for treating CSM. Shi Liang et al.^16^ found that the fusion segment height, cervical Cobb angle, and JOA scores of CSM patients treated with ACDF were significantly improved; Kong Weijun et al.^17^ found that their VAS and JOA scores were significantly improved. In recent years, the focus of clinical ACDF for CSM has shifted to implant materials and implantation methods, and it has been found that different implant materials and implantation methods significantly impact the surgical outcome of ACDF and the prognosis of CSM.

The intervertebral space height, angle and cervical Cobb angle are relatively key measurement parameters of ACDF. ACDF surgery can reconstruct the physiological curvature of the cervical vertebra and restore the sagittal balance of the cervical vertebra to the greatest extent possible, so as to achieve the purpose of treatment. In this study, the Cobb angle of patients in both groups was significantly increased postoperative, with patients in the 3D group seeing a significantly greater Cobb angle than the control group, indicating that 3D Cage could better restore and maintain the postoperative curvature of the cervical vertebra. According to Igarashi et al.^18^, the subsidence rate of the PEEK Cage was lower than that of titanium alloy cages, but the difference was significant only when the cage height was above five mm. Meanwhile, Noordhoek et al.^19^ found no significant difference between PEEK and titanium alloy cages in terms of postoperative intervertebral space height loss. In this study, the postoperative height and angle of the intervertebral space in the 3D group were significantly higher than those in the control group, suggesting that 3D Cage could better restore and maintain the postoperative intervertebral space height and angle of the cervical vertebra.

The analysis suggests that 3D Cage comes with a porous structure that reduces the elastic modulus of the cage, increases the contact area after bone ingrowth, and increases cage stability. Therefore, we believe that 3D Cage is more conducive to maintaining the intervertebral space height and angle after cervical surgery than PEEK Cage.^20^ In terms of fusion rate, there were four patients in the control group who did not achieve bone fusion, while all patients in the 3D group achieved bone fusion. This indicates the fusion effect of 3D printed intervertebral fusion cage is better. We believe that this is consistent with the fact that the 3D printed intervertebral fusion cage has a microporous structure that facilitates the growth of osteoblasts, and its rough surface is also conducive to the adhesion, proliferation, and growth of osteoblasts, This enables the fusion device to have more osseous integration with bone tissue, thereby achieving osseous fusion.

### Limitations of this study:

The 3D Cage used in this study is designed and produced based on the physiological and anatomical data of normal subjects, which is a semi-customized product that can only be available for selection in height and angle. Therefore, further research is still needed to confirm the advantages of 3D printing technology and realize the individualized clinical treatment as soon as possible, thus achieving better effects in clinical application.

## CONCLUSIONS

This study demonstrates that the use of a 3D Cage in the ACDF treatment of CSM may boast therapeutic advantages over the conventional PEEK Cage. The clinical efficacy of 3D printed intervertebral fusion cage placement in the treatment of cervical spondylotic myelopathy may be reliable, and it would be better than traditional intervertebral fusion cages in maintaining the height, angle, cervical Cobb angle, and fusion rate of the intervertebral space.

### Authors’ Contributions:

**FL** and **YS:** Designed this study prepared this manuscript, are responsible and accountable for the accuracy or integrity of the work.

**DeZ:** Collected and analyzed clinical data.

**YM:** Performed the statistical analysis and participated in review and its design.
